# Chinese herbal medicine for hypertension complicated with hyperlipidemia: A protocol for a systematic review and meta-analysis

**DOI:** 10.1097/MD.0000000000024345

**Published:** 2021-02-12

**Authors:** Yinan Liu, Yiqing Liu, Jing Yang, Xue Wang, Chengkui Xiu, Yanhong Hu, Dan Wu, Ye Wu, Yan Lei

**Affiliations:** Experimental Research Center, China Academy of Chinese Medical Sciences, Beijing, China.

**Keywords:** Chinese herbal medicine, hypertension complicated with hyperlipidemia, protocol, systematic review

## Abstract

**Background:**

Hypertension combined with hyperlipidemia (HTN-HLP), as a common clinical chronic disease combination, will increase the incidence of cardiovascular and cerebrovascular diseases, increase the occurrence of sudden death and other adverse events. At present, the commonly used therapeutic drugs are mainly combined with antihypertensive drugs and lipid-lowering drugs, which not only have poor compliance, but also have adverse reactions. Currently, traditional Chinese medicine, as a traditional medicine in China, has been applied in clinical practice for thousands of years and has rich clinical experience in treating HTN-HLP. However, there is no systematic evaluation of the efficacy, safety and improvement of patients’ quality of life. This systematic review and meta-analysis will assess studies of the effects and safety of Chinese herbal medicine (CHM) for HTN-HLP patients.

**Methods:**

We will search PubMed, Embase, Cochrane Central Register of Controlled Trials, Web of Science (ISI), China National Knowledge Infrastructure, Wan fang Database, Chinese Scientific Journals Full-Text Database (VIP) and China Biological Medicine Database from the time when databases were established to 01, February 2021. After a series of screening, randomized controlled trials (RCTs) will be included related to CHM for HTN-HLP. Two researchers will assess the RCTs through the Cochrane bias risk assessment tool. And the evidence grade of the results will be evaluated by GRADEprofiler software.

**Results:**

This study will provide a reliable evidence for the efficiency of antihypertensive and reducing blood lipids of CHM for HTN-HLP.

**Conclusion:**

We will summarize the methods and provide sufficient evidence to confirm the efficacy and safety of CHM for HTN-HLP.

**INPLASY registration number:**

INPLASY2020110144.

## Abstract 2

Supplemental Digital Content is available in the text

## 1 Introduction

Cardiovascular disease (CVD) is the leading cause of death globally, accounting for nearly one-third of all deaths.^[1]^ Hypertension and hyperlipidemia are the main causes and risk factors of CVD. When hypertension and hyperlipidemia coexist, the risk of CVD is greatly increased.^[2,3]^ Epidemiological studies have shown that hypertension complicated with hyperlipidemia (HTN-HLP) is the most common combination of chronic diseases among medical insurance beneficiaries in the United States, hypertension and hyperlipidemia are often complicated in middle-aged and elderly patients.^[4]^ Among patients over 65 years of age, 56% were male and 53.5% were female.^[5]^ In China, at least 23.2% of adults suffer from hypertension and 41.9% suffer from hyperlipidemia. The proportion of patients with dyslipidemia who also have hypertension is 2.1 times higher than those who are normal hypertension.^[6]^ HTN-HLP has created an enormous burden on global public health.

Hypertension is associated with increases in the risks of myocardial infarction, heart failure, ischemic or hemorrhagic stroke, and end-stage renal disease.^[7,8]^ Hyperlipidemia is a lipid metabolism disorder characterized by decreased serum high-density lipoprotein (HDL-C) or elevated serum total cholesterol (TC), triglyceride (TG), and low-density lipoprotein (LDL-C).^[9]^ Studies have shown that HTN-HLP can lead to endothelial dysfunction of coronary arteries and impair endothelium-dependent vasodilation and automatic regulation of cerebral and optic nerve head blood flow.^[10]^ Therefore, the simultaneous control of blood lipid and blood pressure is a problem worthy of clinical attention. Currently, antihypertensive drugs and statins are commonly used in clinical treatment. However, the traditional treatment regimen has poor compliance and often causes adverse reactions (including gastrointestinal events, musculoskeletal pain, respiratory infection, and headache) and other problems.^[8,11,12]^ Not only that, some kinds of antihypertensive drugs may cause metabolic changes that lead to increased insulin resistance and more pronounced dyslipidemia,^[13]^ which make the blood pressure and lipid of HTN-HLP patients unable to reach the ideal state. Despite continued progress in drug development, the prevalence rates worldwide indicate the need to find new, powerful treatments.^[14]^

For thousands of years, traditional Chinese medicine (TCM) has accumulated a lot of clinical experience in the treatment of diseases. Whether acupuncture, cupping, massage, moxibustion, or Chinese herbal medicine (CHM) are widely used in clinical practice so far. Clinical studies show that CHM has a good effect on HTN-HLP. Recent studies have found that CHM combined with western medicine to treat HTN-HLP can create a synergistic effect. In addition, it is superior to the treatment effect of western medicine only in controlling blood pressure and blood lipid, reducing blood hyperviscosity, and promoting the regression of arterial plaque. It can significantly reduce the incidence of cardiovascular and cerebrovascular events with high safety and no obvious adverse reactions.^[15]^ Although clinical studies have shown the application value of CHM, there is a lack of direct evidence to prove the efficacy of CHM in the treatment of HTN-HLP. Therefore, this study aims to conduct systematic review and meta-analysis on randomized controlled trial (RCT) studies of CHM treatment of HTN-HLP through PICOS principles, so as to provide strong evidence for clinical practice.

## 2 Methods

### 2.1 Study registration

This protocol for systematic review was registered on INPLASY platform (https://inplasy.com/inplasy-2020-11-0144/). The registration number is INPLASY2020110144 and the DOI number is 10.37766/inplasy2020.11.0144. In addition, this protocol is structured according to the Preferred Reporting Items for Systematic Reviews and Meta-Analyses Protocols (PRISMA-P) statement guidelines.

### 2.2 Eligibility criteria

Five main factors of PICOS were used for this research: Participant (P); Intervention (I); Comparator (C); Outcome (O); Study design (S).

### 2.3 Inclusion criteria for study selection

#### 2.3.1 Types of studies

We will give preference to RCTs which investigated clinical efficacy and safety of CHM for HTN-HLP. There are no restrictions on methodological quality of RCTs, follow-up, or publication status.

#### 2.3.2 Types of participants

The adult patients (18 years of age and older) were diagnosed as HTN-HLP. HTN should be confirmed according to the “2018 Chinese guidelines for the management of hypertension”^[16]^ and HLP should be confirmed according to the standard diagnostic criteria including the “Guidelines for the prevention and treatment of dyslipidemia in Chinese adults (2016).”^[17]^ There will be no limited to gender, ethnicity and country. Children, pregnant women, patients with secondary hypertension and combined with other complications will be excluded.

#### 2.3.3 Types of intervention and control

We will consider the intervention used clinically common CHM, like Huanglian Wendan Decoction, Banxia Baizhu Tianma Decoction, Xuefuzhuyu Decoction, Huanglian jiedu decoction, and Tianma Gouteng decoction, which were used alone or in combination. There is no limit to duration, dose, or route of administration. Treatments in the control group can be conventional pharmacotherapy, other CHM, other kinds of TCM like acupuncture, or no additional intervention to usual care. If combined treatment of CHM and other therapy were used in the experimental group, the same therapy must be used in the control group.

#### 2.3.4 Types of outcome measures

##### 2.3.4.1 Primary outcomes

- Blood pressure: including systolic blood pressure (SBP), diastolic blood pressure (DBP);
- Blood lipids: including total cholesterol (TC), TG, LDL-C, HDL-C, apolipoprotein A, apolipoprotein B, and so on;
- TCM Symptom Score;
- Total clinical efficacy rate: According to the Guiding Principles for Clinical Research of Drugs^[18]^: markedly effective: diastolic blood pressure (DBP) reached the normal range and decreased >10 mm Hg or DBP did not fall to normal, but it had decreased 20 mm Hg; TC decreased ≥20% or TG decreased ≥40% or HDL-C increased ≥0.26 mmol/L; effective: DBP reached the normal range and decreased <10 mm Hg or DBP decreased by 10 to 19 mm Hg, but did not reach the normal range or SBP decreased >30 mm Hg; TC decreased by 10% to 20% or TG decreased by 20% to 40% or HDL-C increased by 0.10 to 0.26 mmol/L; invalid: did not meet the above criteria. In this study, clinical total effective rate = [(total number of patients-invalid)/total number of patients]×100%.

##### 2.3.4.2 Secondary outcomes

- Adverse reactions;
- 24-hour ambulatory blood pressure;
- Results of echocardiography;
- Quality of life measure like Rating of Activities of daily living, Perceived Exertion Scale, Short Form-36 (SF-36), EQ-5D-5L, and other outcomes;

### 2.4 Exclusion criteria

Animal studies, duplicated data, incomplete data, incorrect data that cannot be extracted or the full text cannot be obtained after contacting original authors.

### 2.5 Search strategy

We will systematically search the published reports on RCTs throughout 8 databases: PubMed, Embase, Cochrane Central Register of Controlled Trials, Web of Science (ISI), China National Knowledge Infrastructure, Wan fang Database, Chinese Scientific Journals Full-Text Database (VIP), and China Biological Medicine Database from the time when databases were established to 01, February 2021. No restriction on publication status was preset. Otherwise, we will also manually retrieve literature from the WHO Trial Register, Google Scholar, Chinese Clinical Trial Register, Baidu, and other search engines to acquire other unpublished articles. Search terms will generally consist of 3 groups: Clinical conditions: HTN-HLP like “Hypertension,” “High Blood Pressure,” “Hyperlipidemia,” and “Dyslipidemia”; types of intervention: CHM such as “Huanglian Wendan Decoction,” “Banxia Baizhu Tianma Decoction,” “Xuefuzhuyu Decoction,” “Huanglian jiedu decoction,” and “Tianma Gouteng decoction”; study type: randomized controlled trial. The search strategy performed in PubMed is presented in Appendix A, http://links.lww.com/MD/F608.

### 2.6 Study selection and data extraction

**Figure 1:**
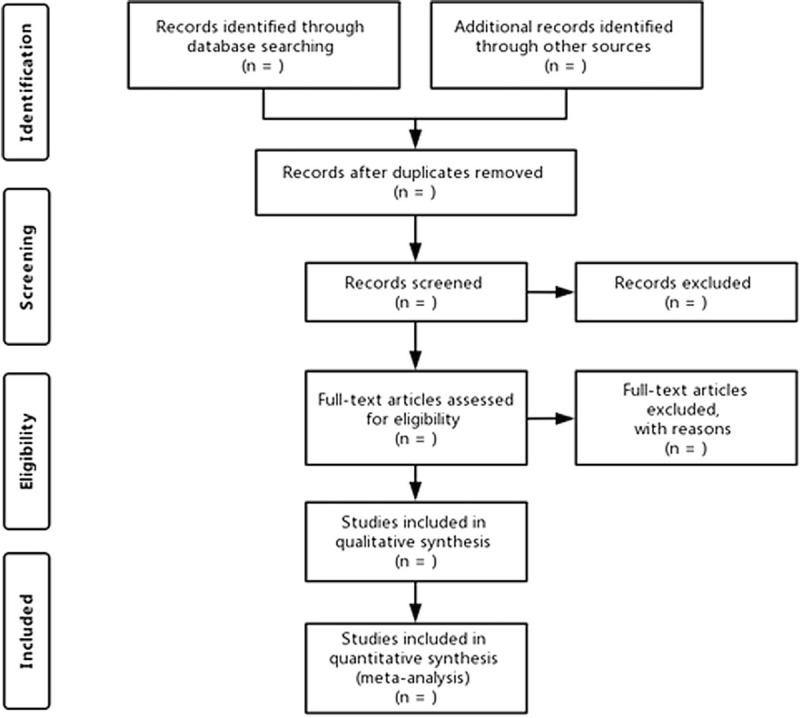
PRISMA flow diagram.

As shown in Figure 1, 2 independent researchers will screen the literature according to the inclusion and exclusion criteria, eliminate duplicate articles through EndNote (version 17.8.0.11583), conduct preliminary screening by reading the title and abstract to exclude the literature that does not meet the inclusion criteria. After reading full texts to make final choice, the details and the number of data studies like basic research information, research methods, intervention and control measures, and results will be extracted independently by 2 researchers using a standardized data abstraction form. Any disagreement over the eligibility of particular studies will be resolved through discussion with a third researcher. Information of the eligible studies was extracted by 2 researchers independently using a standardized data extraction form. The standard data extraction form will include at least the following items: basic information of the studies: title, author details, and publication time; basic characteristics of the patients: age, gender, sample size, diagnosis; basic characteristics of the studies: study design, recruitment strategy, methodological quality, interventions in the experimental and control group, compositions, dosage, duration of treatment, administration methods; primary and secondary outcome measures. We will contact the corresponding authors by email and telephone number for additional information if necessary.

### 2.7 Quality assessment

Two researchers will independently assess the risk of bias in the eligible studies through the risk assessment tool for bias in the Cochrane Handbook version 5.1.0.^[19]^ The evaluation is mainly made from 7 aspects: random sequence generation (selection bias); allocation concealment (selection bias); blinding of participants and personnel (performance bias); blinding of outcome data (attrition bias); incomplete outcome data (attrition bias); selective reporting (reporting bias); other biases. And it evaluates the 7 aspects according to “low risk,” “not clear,” and “high risk.”

### 2.8 Evidence synthesis for RCTs

#### 2.8.1 Meta-analysis

Meta-analysis will be processed by Review Manager software (Version 5.3, Copenhagen: The Nordic Cochrane Center, 2014). Dichotomous outcome was expressed by risk ratio (RR), continuous data as mean difference (MD), both with 95% confidence interval (CI). *P* < 0.05 was considered to indicate a statistically significant result. The heterogeneity test was determined by combining the *Q* value test with the *I*^*2*^. When *P* > 0.1 and *I*^*2*^ < 50%, there was no heterogeneity, and the fixed-effect model was used for analysis. If *I*^*2*^ > 50%, there is heterogeneity,^[20]^ subgroup analysis will be performed to investigate the sources of heterogeneity. If the subgroup analysis results show that there is sufficient similarity between the subgroups (*P* > 0.1, *I*^*2*^ < 50%), the fixed-effect model will be used for meta-analysis; otherwise, if there is a statistical heterogeneity but no clinical heterogeneous temperament between subgroups, the random-effects model will be conducted. The subgroup or sensitivity analyses, or only descriptive analyses, will be performed if significant clinical heterogeneity exists.

#### 2.8.2 Subgroup analysis

When conducting meta-analysis, for each outcome studies will be grouped according to: the type of treatment (Huanglian Wendan Decoction, Banxia Baizhu Tianma Decoction, Xuefuzhuyu Decoction, Huanglian jiedu decoction, and Tianma Gouteng decoction, et al); the comparator (no treatment, general treatment, or other kinds of CTM).

#### 2.8.3 Sensitivity analysis

Sensitivity analysis will be performed according to clinical factors (age, gender, Chinese medicine syndrome), intervention method (Huanglian Wendan Decoction, Banxia Baizhu Tianma Decoction, Xuefuzhuyu Decoction, Huanglian jiedu decoction, and Tianma Gouteng decoction), or methodological heterogeneity (sample size, risk of bias).

#### 2.8.4 Publication bias

We will assess publication bias by the visual asymmetry on a funnel plot, if at least 10 trials are included in the study.^[21]^ If the funnel plot is asymmetrical and does not have an inverted funnel shape, publication bias may occur.

#### 2.8.5 Grading the quality of evidence

We will assess the quality of evidence through GRADEprofiler Version 3.6 software (The GRADE Working Group, 2010). Based on “Risk of bias,” “Inconsistency,” “Indirectness,” “Imprecision,” “Publication Bias,” and other aspects, we assess the evidence as 4 levels: high quality, moderate quality, low quality, and very low quality. Two researchers will evaluate certainty of evidence separately. If there is a dispute, we will have a discussion to solve it.

### 2.9 Ethics

Since systematic review and meta-analysis does not involve the medical research on human subjects and the collection of private information, this research does not require ethical approval.

## 3 Discussion

With the increasing incidence of cardiovascular and cerebrovascular diseases year by year, hypertension combined with hyperlipidemia has become a combination of diseases affecting global health. In recent years, the clinical studies on HTN-HLP have found that CHM has been extremely effective in controlling blood pressure, improving the metabolism of serum lipids, reducing adverse reactions, and improving the quality of life. However, there is a lack of systematic review and meta-analysis on the efficacy and safety of TCM in the treatment of HTN-HLP. Therefore, our purpose is to summarize the systematic review and meta-analysis to provide sufficient evidence for the efficacy of TCM in the treatment of hypertension complicated with hyperlipidemia, and summarize the TCM therapy for HTN-HLP. However, there are some limitations in this study. First, we limit the language to Chinese and English clinical studies, and there may be a lack of research on other languages. Second, our study only involved adults over 18 years of age, and there is a lack of research on children and pregnant women. Finally, different CHM dosage and treatment course may lead to great heterogeneity.

## Author contributions

**Conceptualization:** Yinan Liu, Yiqing Liu, Yan Lei.

**Data curation:** Yanhong Hu, Yiqing Liu, Dan Wu, Ye Wu.

**Methodology:** Yinan Liu, Jing Yang, Chengkui Xiu, Xue Wang.

**Software:** Yinan Liu.

**Supervision:** Jing Yang, Yan Lei.

**Writing – original draft:** Yinan Liu, Yiqing Liu, Dan Wu, Ye Wu.

**Writing – review & editing:** Yinan Liu, Yiqing Liu, Dan Wu, Ye Wu.

## Abbreviations

Abbreviations: CHM = Chinese herbal medicine, CI = confidence interval, CNKI = China National Knowledge Infrastructure Database, CVD = cardiovascular disease, DBP = diastolic blood pressure, GRADE = Grading of Recommendations Assessment, Development and Evaluation, HDL-C = high-density lipoprotein, HTN-HLP = hypertension combined with hyperlipidemia, ISI = Web of Science, LDL-C = low-density lipoprotein, MD = mean difference, PRISMA-P = Preferred Reporting Items for Systematic Reviews and Meta-Analyses Protocols, RCTs = randomized controlled trials, SBP = Systolic Blood Pressure, SF-36 = Short Form-36, TC = total cholesterol, TCM = traditional Chinese medicine, TG = triglyceride, VIP = Chinese Scientific Journals Full-Text Database.

## References

[1] Hong Kimberly N Fuster Valentin Rosenson Robert S. How low to go with glucose, cholesterol, and blood pressure in primary prevention of CVD. J Am Coll Cardiol 2017;70:2171–85. 29050566 10.1016/j.jacc.2017.09.001

[2] Hasan R Kamal Ayeesha K Morris Pamela B. Mobile Health (mHealth) technology for the management of hypertension and hyperlipidemia: slow start but loads of potential. Curr Atheroscler Rep 2017;19:12. 28210974 10.1007/s11883-017-0649-y

[3] Krone W Müller-Wieland D. Hyperlipidaemia and hypertension. Baillieres Clin Endocrinol Metab 1990;4:833–50. 2082908 10.1016/s0950-351x(05)80081-3

[4] Castaño G Más R Fernández JC. Effects of policosanol on older patients with hypertension and type II hypercholesterolaemia. Drugs R D 2002;3:159–72. 12099160 10.2165/00126839-200203030-00004

[5] Zhou B Ren C Zu L. Elevated plasma migration inhibitory factor in hypertension-hyperlipidemia patients correlates with impaired endothelial function. Medicine (Baltimore) 2016;95:e5207. 27787379 10.1097/MD.0000000000005207 PMC5089108

[6] Jun HS Saem JH Cho J-M. Efficacy and safety of triple therapy with telmisartan, amlodipine, and rosuvastatin in patients with dyslipidemia and hypertension: the jeil telmisartan, amlodipine, and rosuvastatin randomized clinical trial. Clin Ther 2019;41: 233–248.e9. 10.1016/j.clinthera.2018.12.008 30665829

[7] Abell Jessica G Kivimäki M Dugravot A. Association between systolic blood pressure and dementia in the Whitehall II cohort study: role of age, duration, and threshold used to define hypertension. Eur Heart J 2018;39:3119–25. 29901708 10.1093/eurheartj/ehy288 PMC6122131

[8] Azizi M Rossignol P Hulot J-S. Emerging drug classes and their potential use in hypertension. Hypertension 2019;74:1075–83. 31495277 10.1161/HYPERTENSIONAHA.119.12676

[9] Ducharme N Radhamma R. Hyperlipidemia in the elderly. Clin Geriatr Med 2008;24: 471–87, vi. 10.1016/j.cger.2008.03.007 18672183

[10] Hashimoto R Sugiyama T Ubuka M. Impairment of autoregulation of optic nerve head blood flow during vitreous surgery in patients with hypertension and hyperlipidemia. Graefes Arch Clin Exp Ophthalmol 2017;255:2227–35. 28940022 10.1007/s00417-017-3788-5

[11] Liu M Zhang Q Jiang S. Warm-needling acupuncture and medicinal cake-separated moxibustion for hyperlipidemia: study protocol for a randomized controlled trial. Trials 2017;18:310. 28693531 10.1186/s13063-017-2029-x PMC5504830

[12] Samantha K. Epidemiology and management of hyperlipidemia. Am J Manag Care 2017;23:S139–48. 28978219

[13] Lithell H. Hypertension and hyperlipidemia. A review. Am J Hypertens 1993;6:303S–8S. 8297536 10.1093/ajh/6.11.303s

[14] Amela J Yvan D. EU-CardioRNA COST Action (CA17129), noncoding RNAs in hypertension. Hypertension 2019;74:477–92. 31352819 10.1161/HYPERTENSIONAHA.119.13412 PMC6686966

[15] Xue Y Yang X Wu D. Observation on the curative effect of Huanglian Jiedu Decoction combined with Rosuvastatin on hypertension complicated with hyperlipidemia. Hebei Pharmaceutical 2012;40:3269–72.

[16] Writing Group of the 2018 Chinese Guidelines for the Management of Hypertension. 2018 Chinese guidelines for the management of hypertension. Chin J Cardiovasc Med 2019;24:24–56.

[17] Zhu J Gao R Zhao L. Guidelines for prevention and treatment of dyslipidemia in Chinese adults (2016 revised edition). Chin J Circ 2016;16:15–35.

[18] Zheng X. Guidelines for the Clinical Research of Chinese Medicine New Drugs [M]. Beijing: China Medical Science Press; 2002.

[19] Higgins JPT Green S. Cochrane Handbook for Systematic Reviews of Interventions Version 5.1.0 (updated March 2011). The Cochrane Collaboration, 2011.

[20] Higgins JP Thompson SG Deeks JJ. Measuring inconsistency in meta-analyses. BMJ 2003;327:557–60. 12958120 10.1136/bmj.327.7414.557 PMC192859

[21] Sterne JAC Egger M Moher D. Higgins JPT Green S. Chapter 10: addressing reporting biases. John Wiley & Sons, Cochrane Handbook for Systematic Reviews of Interventions. Chichester, UK:2008.

